# Phosphoproteomics: Advances in Research on Cadmium-Exposed Plants

**DOI:** 10.3390/ijms252212431

**Published:** 2024-11-19

**Authors:** Deyvid Novaes Marques, Fernando Angelo Piotto, Ricardo Antunes Azevedo

**Affiliations:** 1 Department of Genetics, Luiz de Queiroz College of Agriculture (ESALQ), University of São Paulo (USP), Piracicaba 13418-900, São Paulo (SP), Brazil; 2 Boyce Thompson Institute, Cornell University, Ithaca, NY 14853, USA

**Keywords:** abiotic stress response, cadmium toxicity, crops, detoxification, heavy metal, phosphoproteomics, protein phosphorylation, plant tolerance, proteomic approaches

## Abstract

With the increasing concern on heavy metal contamination in agriculture and other environmental settings, unraveling the mechanisms of cadmium (Cd) tolerance and response in plants has become highly important. Ongoing plant Cd research over the years has focused on strategic and relevant aspects, including molecular, biochemical, and physiological processes. From this perspective, phosphoproteomics appears to be an innovative and powerful approach to investigating plant responses to Cd stress. Here, we summarize progress in plant Cd research across different plant species regarding large-scale phosphoproteomic investigations. Some studies revealed major proteins participating in detoxification, stress signaling, and metabolism, along with their regulation through phosphorylation, which modulates the plant’s defense against Cd. However, many pathways remain unexplored. Expanding these studies will help our ability to alleviate Cd stress and provide further information concerning involved mechanisms. Our purpose is to inspire researchers to further explore the use of phosphoproteomics in unraveling such complex mechanisms of Cd tolerance and response across various plant species, with the ultimate aim of enhancing strategies for mitigating Cd stress in agriculture and polluted environments.

## 1. Cadmium in Plants and the Phosphoproteomic Context

Cadmium (Cd), a significant abiotic stress factor, is among the most toxic heavy metals, which poses significant challenges to plant metabolism and agricultural safety. Cd is non-essential in plants and animals, and even at low concentrations it may severely affect physiological processes, thus being highly hazardous when entering the food chain (reviewed by Marques et al. [1,2]). It is released into the environment both through natural and anthropogenic sources, including mining activities, industrial releases into the air, and phosphate-based fertilizers. According to the existing literature [3,4], Cd has been reported to exist in several forms of chemical compounds; cadmium sulfide (CdS) is a prevalent form of this metal in nature. The formation of Cd(CN)_4_^2^⁻ and Cd(NH_3_)_6_^4^⁻ has been known to derive from the combination of CdS with cyanide and ammonia, respectively [3]. This diversity indicates a profound influence on the behavior of Cd in the environment and its interaction with biological systems. After plants uptake Cd, it interferes with important physiological processes within the plants (some of them reviewed by Clemens et al. [5] and Clemens and Ma [6]). Thus, the understanding of how plants respond to Cd stress and elucidating related tolerance mechanisms are important prerequisites in basic plant physiology regarding Cd research and developing effective strategies for Cd mitigation.

Understanding the dynamics of cell signaling in response to Cd exposure is relevant to delineate the molecular mechanisms that allow plants to cope with and adapt to Cd-induced stress. According to the review by Vitelli et al. [7], analysis of how such signaling pathways work under Cd stress provides critical insight into the capability of the plant to temper the toxic effects of Cd and maintain cellular function.

Protein phosphorylation represents one of the major and crucial signaling regulatory mechanisms in plant responses to various types of stress, including Cd exposure. Such a process includes the addition of phosphate groups to proteins, thus changing their activity, stability, or ability to interact with other cellular components. This post-translational modification serves as a kind of molecular switch that turns on or off multiple signal pathways that are crucial for plant development, growth, and adaptation against environmental challenges such as toxicity caused by heavy metals. Importantly, protein phosphorylation turns on the detoxification mechanisms and stress signaling pathways of plants; this in turn ameliorates the detrimental action of stress [8].

Proteomics—the field of research focused on all proteins present in a biological sample, particularly with regard to various relevant aspects, including their functions, structures, and interactions in biological systems—has gained the limelight as an effective tool in unraveling the molecular basis of plants’ responses to stressors. The information on the set of proteins expressed under stress conditions could enable the identification of proteins important in the defense and adaptation processes of the plant. Proteomic approaches have greatly improved our knowledge on how plants cope with Cd stress through the identification of proteins taking part in signaling, metabolic, and detoxification processes [9]. We investigated Cd-exposed plants in several studies, which included a suite of proteomic approaches to point out molecular mechanisms of tolerance to Cd [1,10] and our pioneer phosphoproteomic investigation using tomato (*Solanum lycopersicum*) plants [11].

Phosphoproteomics, a specialized proteomics field, has involved insights on the identification and quantification of phosphopeptides, as well as the phosphorylation status of proteins, which is one critical aspect in the regulation of cellular processes under stress. Therefore, it enabled the investigation into how phosphorylation dynamically regulates proteins during stress responses. This makes phosphoproteomics of particular importance for studying abiotic stresses, considering that phosphorylation controls major signal transduction pathways and metabolic adjustments that enable plants to tolerate stress [12]. Identifying the specific phosphorylation events that occur in Cd allows the use of phosphoproteomics to investigate the molecular mechanism involving tolerance and detoxification.

Although some studies using proteomic and phosphoproteomic approaches have been conducted on Cd stress responses among plant species, phosphoregulation is still in the early stages of study. Much remains to be explored in terms of specific proteins and pathways regulated by phosphorylation under Cd exposure. Thus, detailed studies of phosphorylated proteins in plants exposed to Cd will reveal more about how plants use strategies for Cd detoxification and mitigation. Clearly, there is a need for a better understanding of how Cd exposure modulates the function of proteins through phosphorylation. Herein, we summarize information on these phosphoproteomic investigations and also provide perspectives on the current state and future directions of this field.

## 2. Insights into Protein Phosphorylation in Plants Under Cadmium Exposure

Some of the mechanisms involved in Cd-induced protein phosphorylation without using large-scale phosphoproteomic approaches have been at least partially explained by various studies across different plant species. For instance, Reddy and Prasad [13] determined that changes occur in protein phosphorylation in rice (*Oryza sativa*), where it was found that Cd preferentially increased the phosphorylation of a 68 kDa protein (p68) while decreasing the phosphorylation level of another heat shock-related cognate protein, hsc70. In another investigation, a heat shock associated with Cd treatment inducing protein phosphorylation in soybean (*Glycine max*) seedlings has been shown [14]. Furthermore, the influence of Cd on nitrate assimilation in bean (*Phaseolus vulgaris*) through modifications of the activation states of nitrate reductase, as well as the related role of protein phosphorylation in metabolic regulation, has been investigated [15]. Some authors have analyzed protein phosphorylation events and reported the activation of some kinases (including mitogen-activated protein kinases (MAPKs) and myelin basic protein kinases) in response to Cd in alfalfa (*Medicago sativa*) [16] and rice plants [17] in a heavy metal dose-dependent manner.

Further information touches upon the capability of brassinosteroids to increase the phosphorylation level of plasma membrane H^+^-ATPase under Cd stress, as reported by Jakubowska and Janicka [18]. The investigation conducted by Ma et al. [19] revealed a protein kinase SOS2L1 (a calcineurin B-Like-interacting protein kinase) in apple (*Malus domestica*), which controls malate excretion and interacts with the malate transporter MdALMT14. The phosphorylation-induced stability of MdALMT14 under Cd exposure was also noted [19].

Zhang et al. [20] pointed out the role of calcium-dependent protein kinases in regulating Cd tolerance in *Arabidopsis*; Du et al. [21] identified and demonstrated a receptor-like kinase (TaWAK20) in wheat (*Triticum aestivum*), which is induced by Cd treatment. Taken together, these studies have highlighted the importance of phosphorylation events as a post-translational change in mediating plant responses to Cd stress and the relevance of non-omic molecular and biochemical approaches for the improvement of this field of research. Phosphoproteomics in combination with other approaches will provide more detailed information about complex signaling networks regulating plant responses to Cd stress. These integrative approaches may point to key phosphorylation events associated with the modulation of Cd tolerance, thus identifying genetic targets for use in biotechnological applications. Additionally, the interaction between different signaling pathways involving kinases and plant hormones will be understood and lead to better resilience and improved plants to Cd exposure.

## 3. Phosphoproteomics in Cadmium-Exposed Plants

### 3.1. Investigations of Different Plant Species

Our research group carried out a pioneering comparative phosphoproteomic analysis of tomato genotypes with contrasting tolerance to Cd, identifying thousands of phosphopeptides [11]. This was the first large-scale study of its kind on tomato. Cd treatment led to an increased difference in phosphorylation status between the Cd-tolerant and Cd-sensitive tomato genotypes in relation to proteins associated with major functions. This included phytohormones-related proteins, kinases and phosphatases, and transcription factors, and heat shock proteins (HSPs). Other protein groups found in this tomato research included ABC (ATP-Binding Cassette) transporters and HSPs, which are proposed as important contributors to the Cd detoxification process, potentially facilitating Cd sequestration into vacuoles by working synergistically or in parallel with phytochelatins (PCs) [22] and preventing Cd-induced protein misfolding [23], respectively. These observations suggest that detoxification, stress signaling, and transcriptional pathways—essential for maintaining normal plant homeostasis under Cd stress—are regulated to some extent by phosphorylation in tomato.

Regarding rice plants, large-scale phosphoproteomic studies have reported that proteins with altered phosphorylation status under Cd treatment were involved in a wide range of cellular processes (e.g., signal transduction and transcriptional regulation), as well as ABC transporters and HSPs [24]. Protein phosphorylation has also been reported as a contributing factor in the modulation of rice’s oxidative stress response and protection against Cd-induced cell damage [24]. Another comparative study by Fang et al. [25] examined the differential accumulation of phosphorylated proteins between Cd-tolerant (D28) and Cd-sensitive (D69) rice lines and identified proteins associated with key metabolic processes—such as carbon metabolism, proteolysis, DNA repair, RNA helicases, and transcription—in the tolerant one. These findings suggest that protein phosphorylation-related events also contribute to the fine-tuning of metabolism to detoxify Cd related to the proteins from these pathways in rice [25].

Other authors also conducted a phosphoproteomic investigation and observed that fructose 1,6-bisphosphate aldolase (PvFBA1), as well as proteins related to various metabolic pathways (e.g., carbohydrate and energy metabolism), were differentially phosphorylated in Seashore paspalum (*Paspalum vaginatum*) plants under Cd exposure [26]. Considering the potential of this perennial turfgrass for use in phytoremediation, these findings provide additional information on the applicability of phosphoproteomics. Altogether, these findings complement the findings from key food crop species mentioned above and indicate the potential of phosphoproteomics research beyond Cd mitigation mechanisms within a crop safety context. Within this context, strategic tools to increase the knowledge of Cd-tolerant plants that could eventually be used in the future with enhanced metal hyperaccumulation to advance environmental cleanup are also relevant aspects to be further explored.

### 3.2. Nitrogen Supplementation Modulating Protein Phosphorylation in Cadmium-Exposed Plants

Some authors studied the phosphoproteomic responses of poplar plants to Cd stress and the consequences of supplementation with N, respectively. The supplementation of nitrogen (N) was found to enhance plant resistance to Cd stress by modifying key signaling pathways and enhancing stress response mechanisms in such previous studies [27,28]. He et al. [27] identified significantly enriched phosphorylated protein sites and differentially expressed kinases and underlined the role of N application in increasing protein phosphorylation and boosting key proteins in sugar synthesis pathways, such as UDP-glucose dehydrogenase and sucrose-phosphate synthase.

Huang et al. [28] found that, due to Cd stress, some proteins with phosphosites were differentially regulated (e.g., proteins involved in the cellular response to abscisic acid (ABA) stimulus and those with calmodulin protein kinase activity), with some being upregulated and others downregulated. However, N + Cd treatment induced changes in such phosphorylation patterns, generally counteracting the downregulation caused by Cd stress. This shift suggests that N plays a role in modulating protein phosphorylation status under Cd stress, likely contributing to nitrogen’s protective effects [28].

Potential opportunities lie in a more comprehensive phosphoproteomic view along with N-involved responses in more plant species. This includes investigations focused on N availability [29] and nitric oxide (NO) [30], which have been involved in Cd-induced responses and Cd mitigation in plants. The combination of the effect of N on plant metabolism and stress signaling with phosphoproteomic aspects and N metabolism-related molecules in multiple plant species might help to unravel more mechanisms underlying the wide array of N implications under various experimental conditions.

**Figure 1:**
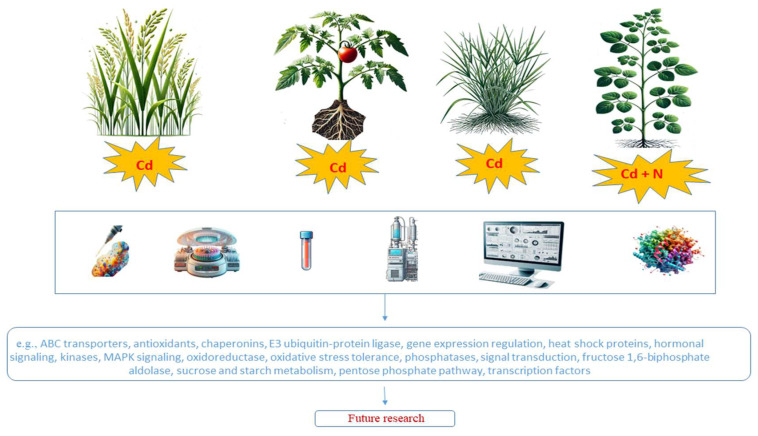
Graphical overview of different plant species under Cd exposure, highlighting exemplary differentially regulated molecules and mechanisms identified in phosphoproteomic studies.

In Figure 1, we present a graphical overview of plant species studied, highlighting exemplary differentially regulated molecules identified in phosphoproteomic analyses under Cd exposure.

## 4. Physiological and Biochemical Insights Complement Results of Phosphoproteomic Analyses in Cadmium-Exposed Plants

A holistic understanding of how plants respond to Cd stress requires the integration of proteomic and phosphoproteomic data with physiological and biochemical analyses. Indeed, the integration of phosphoproteomic and physiological data points to a future research direction where a more complete understanding of plant responses to Cd stress will emerge. Omics-based studies provide essential information on molecular changes that lead to the activation of physiological mechanisms in response to Cd stress across different experimental conditions (Table 1).

The different aspects of this will now be discussed in light of various perspectives as follows:

1. Our research group has been undertaking the challenge of unraveling the physiological responses of the tomato genotypes used in the phosphoproteomic approaches mentioned above [11]. Thus, this encompasses the studies covering different approaches including those involving oxidative stress parameters, other proteomic approaches, antioxidant enzyme activities, and the accumulation of Cd and nutrients in plant tissues (see insights and information reviewed in Marques et al. [1,2]). In addition, these physiological data are well-positioned to provide an overview of the broad context of mechanisms underlying Cd tolerance, pointing out that several levels of analysis have to be considered to fully understand tomato responses to Cd.
2. He et al. [27] demonstrated that N supplementation in poplar plants mitigated the Cd-induced loss of chlorophyll and increased soluble sugar content. Such physiological improvements are associated with membrane stability and ion balance during heavy metal stress. Moreover, N augmented the activities of key antioxidant enzymes that mitigate oxidative stress imposed by Cd [27]. This demonstrates that N participates not only in supporting photosynthesis but also in enhancing cellular defense mechanisms. Further investigation showed that N supplementation in Cd-exposed poplar plants increases the synthesis and activity of stress-responsive proteins, including HSP70 and peroxidase (POD). These proteins support plants in protein stability maintenance and detoxifying reactive oxygen species (ROS), thereby reducing oxidative damage. It is interesting that N supplementation enhanced Cd uptake, suggesting that poplar plants could use N to cope better with Cd accumulation through an integrated action of more effective detoxification and sequestration mechanisms [28].
3. Studies have demonstrated that applying N to poplar plants under Cd stress leads to an increase in the levels of proteins which play key roles in sucrose and starch metabolism [27,28]. Considering that other authors have reported that different forms of N additions can alleviate stress induced by other heavy metals besides Cd [31], the exploration and phosphoproteomic observation in a multi-stress context is also relevant. Indeed, the potential of phosphoproteomic studies to identify key regulatory proteins and phosphorylation sites involved in plant responses to combined stresses or combined environmental settings, such as heavy metal toxicity and nutrient availability, is thus a potential focus in further investigations. Furthermore, future research could focus on further determining how these changes in sucrose metabolism contribute to Cd tolerance in poplar and other crops.
4. Huang et al. [28] found that exogenous N enhances the efficiency of poplar plants in responding to Cd stress by identifying differentially accumulated proteins in parallel with phosphoproteomic findings, which included translationally controlled tumor proteins (TCTPs). The relevance of TCTPs have been highlighted for its potential and application in plant growth improvement and abiotic stress tolerance [32]; however, the detailed mechanisms are yet to be determined. Even though some phosphoproteomic studies have not confirmed such a differential regulation of TCTP under Cd stress, these results suggest that a study of TCTPs within a phosphoproteomic framework may reveal their specific functions regarding their role in the development of stress tolerance. This is particularly important because TCTP-related mechanisms may differ in different plant species and under specific treatments [32], suggesting diverse phosphorylation patterns may appear together with such responses. More investigations will be required to understand the phosphorylation dynamics in parallel with TCTPs function and other candidates, as well as interactions with other stress-related proteins that could enrich our understanding of the molecular network constituting plant resilience to diverse stresses.
5. In addition to N, the investigation of other elemental treatments is equally relevant in the context of phosphoproteomics and Cd exposure in plants. For example, Cu homeostasis is important for normal plant growth under Cd toxicity [33]. Importantly, the complex interplay of other factors, such as K, Ca, and Mg accumulation, has been reviewed as playing a critical role in modulating signaling pathways under Cd stress [2]. Such elements may therefore alter the physiological and biochemical processes of the plant and provide tolerance or sensitivity mechanisms. Consequently, studies need to be expanded from N to other essential elements so that a comprehensive understanding of the responses of plants to Cd may be made at the phosphoproteomic level.
6. Using a phosphoproteome approach, Fang et al. [25] showed that a Cd-tolerant rice genotype (D28) exhibited increased expression of several phosphorylated proteins involved in key processes potentially related to Cd tolerance. These molecules included proteolytic enzymes, enzymes involved in carbon metabolism, and stress-related proteins such as F-box transcription factors and a DEAD-box ATP-dependent RNA helicase. Either way, further research, incorporating phosphoproteomic analysis to identify phosphorylation changes and status in detoxification-related proteins, is needed to definitively establish the functional connections between these metabolic changes and detoxification mechanisms in Cd-tolerant rice and across different rice genotypes. Other findings indicated that phosphorylated proteins involved in ROS detoxification play an important role in protecting cellular structures against Cd-induced oxidative stress in rice [24]. This adds an important layer of understanding to how rice plants integrate signaling pathways and enzymatic defenses to maintain homeostasis under Cd exposure.
7. Another important approach for understanding Cd stress responses in plants is to investigate protein interactions among differentially phosphorylated proteins, as shown by Zhong et al. [24]. The protein–protein interaction network generated in their study has revealed important kinases and phosphatases and specifically showed that PP2C family phosphatases are central players in response to Cd. These findings suggest ABA signaling pathways may play an important role in Cd detoxification and tolerance. Furthermore, the identification of proteins involved in osmotic stress tolerance, such as SAPK6, along with other pathways, including MAPK and CaMK [24], point to the complex molecular responses to Cd stress that should be explored in further studies by combining protein–protein interaction analysis with phosphoproteomics. Further details on signaling networks responsible for regulating plant resilience against this environmental stress will be valuable.
8. Furthermore, protein phosphorylation plays a key role in hormone-mediated signal transduction pathways. Among them, auxins, cytokinins, brassinosteroids, gibberellin, ABA, and ethylene control such processes with phosphorylation being crucial in modulating the activity of hormone receptors and downstream signaling proteins [8]. Various phosphoproteomic analyses discussed above show that the phenomenology of phosphorylation lies at the core of cellular responses to stress, including those driven by hormones. For example, we identified auxin and ethylene signaling pathways by studying the phosphorylation of proteins in Cd-tolerant tomato genotypes [11]. Proteins in the ethylene biosynthesis pathway of Cd-treated rice plants were differentially phosphorylated as well [25]. Thus, future phosphoproteomic work, in concert with hormonal analyses, will elucidate how Cd stress specifically modifies signaling networks through phosphorylation.
9. Zhong et al. [24] and Marques et al. [11] reported that ABC transporters were induced after Cd stress. Huang et al. [28] reported that exogenous N significantly decreased Cd-induced hydrogen peroxide (H_2_O_2_) and malondialdehyde (MDA) generation, whereas it enhanced the accumulation of glutathione and PCs. Interestingly, the authors identified that ABC transporter proteins accounted for the transporter proteins upregulated by the N + Cd treatment [28]. As shown and previously reviewed [9,34,35,36], ABC proteins have been implicated in the action of PCs in the important modification of Cd tolerance by binding and sequestering heavy metals. Further still, this interplay between phosphorylation dynamics, ABC transporters, and PCs points to an important area of future research.
10. Transcription factors are important in orchestrating gene expression regarding Cd stress tolerance, and their targets include genes responsible for antioxidant defense and metal chelation [37,38]. The contrasting Cd tolerance in plant crop phosphoproteome investigations [11,25] and rice Cd-induced phosphoproteomic response [24] was shown to be related to transcription factors. It would therefore be of interest to determine how phosphorylation changes their activity in response to Cd; hence, future research could focus on how phosphoregulation modulates transcription factor networks.
11. The proteomic investigations and insights achieved so far highlighted the significant impact of Cd exposure on the differential regulation of kinases and/or phosphatases [11,24,25,27,28]. This interplay suggests a crucial regulatory mechanism for maintaining cellular homeostasis under the given heavy metal stress involving such enzymes. This topic warrants further investigation in order to further explore how targeted modulation of these molecules could modulate stress tolerance.
12. Cd-induced changes in the phosphorylation level of PvFBA1 and other potential molecules related to Cd tolerance in *Seashore Paspalum* were reported. In parallel, the ability of Cd to bind to PvFBA1, modulating its phosphorylation, in microscale thermophoresis experiments was also observed [26]. In such investigations, the authors reported the genetic transformation of *Arabidopsis thaliana* using the overexpression of the *PvFBA1* gene and related increases in soluble sugar content levels in transgenic plants [26]. These findings indicate the relevance of further exploring the combination of approaches related to plant physiology and biochemistry and genetic engineering tools (including the use of transgenic plants) within the Cd research context. These integrated strategies and applications in other plant species and experimental settings, along with phosphoproteomic insights, will also be relevant in future studies.
13. Overall, such physiological and biochemical findings broaden our knowledge of Cd tolerance by underlining the intricate interrelationship underlying the complexity of phosphoproteomic-related signaling pathways and molecular strategies involved in plant responses to Cd. Further studies need to be conducted to clarify specific phosphorylation patterns and phosphoproteomic profiling across multifaceted cellular and molecular strategies that have so far been put forward for mitigating Cd toxicity. This involves vacuolar chelation and sequestration, the dynamics of signaling and physiological pathways, investigations corresponding to transcription regulation, transport, accumulation studies, HSPs and chaperones, and antioxidant involvement. The involvement of potential molecules also needs further investigation in these aspects since their functions relating to stress response are not yet established.

## 5. Future Multi-Omics Perspectives

In some investigations involving Cd-exposed plants, proteomics and/or differential gene expression were investigated in parallel with phosphoproteomic parameters [24,25,27,28]. Transcriptomic experiments have shed light on the molecular mechanisms behind the plant’s response to Cd stress, but many of these experiments have been performed without considering accompanying proteomic/phosphoproteomic analysis to evaluate how changes in transcriptional profile relate to protein expression and phosphorylation patterns. Gu et al. [39] conducted a comparative transcriptome analysis in *Medicago sativa* and found that melatonin pretreatment to Cd stress induced the expression of a large number of genes involved in protein folding, sorting, and degradation, as well as those involved in protein phosphorylation in sugar metabolism, along with genes encoding kinases (e.g., hexokinase-1). In the context of response to Cd stress and transcriptomic research, Yu et al. [40] identified the involvement of protein phosphorylation in maize seedlings, with key pathways including those of protein modification and signal transduction and differential expression of genes encoding ATP-dependent 6-phosphofructokinase 3 and MAP3K epsilon protein kinase 1. These studies have underlined how the transcriptional regulation related to protein phosphorylation plays an important role in plant resilience to heavy metal stress and have emphasized that future studies should consider multi-omics approaches to understand such complex interactions.

## 6. Exemplary Studies for Broader Implications of Phosphoproteomics

Exemplary studies, not directly addressing plant Cd exposure but giving valuable information relevant to Cd response, are discussed below. Kohorn et al. [41] examined the phosphoproteomic response of *Arabidopsis thaliana* to oligo-galacturonides, pectin fragments released in response to pathogen attacks. The study that follows highlighted that proteins undergo rapid phosphorylation changes in the WAKs (Wall-Associated Kinases) and MAPK signaling pathways, which also overlap with those activated under Cd stress. It suggests that common phosphorylation mechanisms are used by the plant to respond to the biotic and abiotic stressors, including Cd, supporting its versatility as a general defense strategy.

In an isotopic dimethyl labeling-based quantitative phosphoproteomic study of soybeans, Moradi et al. [42] defined the phosphorylation events that included proteins implicated in calcium signaling and ROS detoxification, both critical components for the management of oxidative stress induced by heavy metals. Though the focus of this study was not exactly on Cd, it highlights phosphorylation as a critical regulator of core stress response pathways that are also activated under Cd stress. These model studies illustrate how phosphoproteomic research, even in non-Cd contexts, stands to offer valuable insights into Cd mitigation mechanisms. These studies extend our knowledge of the phosphorylation networks utilized by plants during their response to a wide array of stressors. They illustrate that some of the phosphorylation events identified in the non-Cd context may also apply in the context of Cd stress responses and provide a basis for further researching Cd tolerance mechanisms in environments under different or multiple stressors.

## 7. Concluding Remarks and Additional Future Directions

1. Phosphoproteomic studies have significantly expanded our knowledge on plant responses to Cd stress through the identification of key phosphorylation sites and events along with regulated proteins that participate in modulating the signaling, metabolic, and detoxification cascades. The identification of conserved phosphorylation events across more plant species opens promising perspectives for breeding Cd-tolerant varieties. These studies not only reveal ways in which Cd tolerance and response pathways operate at the molecular and biochemical level but also provide a firm biological basis upon which crops that can tolerate Cd-contaminated environments could be developed. The knowledge sought and gained from all these aspects will become critical in enhancing the resilience of crops and will go a long way toward fulfilling agricultural production on contaminated soils in a sustainable manner.
2. To fully exploit this information, these individual protein groups would have to be studied in more detail, including their interactions with other proteins. Functional validation of such proteins can demonstrate more specific roles they play in Cd tolerance and response, and hence provide additional and novel targets for breeding or engineering Cd-resistant crops.
3. Furthermore, there is an increasing demand for integrative studies of physiological and biochemical analyses complementary to omics data. Using such diverse approaches will enrich the information obtained from phosphoproteomics as Cd stress induces multiple parallel responses in plants. More comprehensive studies are needed to correlate the molecular responses with the observable physiological characteristics. This may create more encompassing methods for developing Cd-tolerant crops and plant genotypes.
4. Besides the studies involving a large number of plant species and experimental settings in phosphoproteomics, Cd tolerance and sensitivity mechanisms still lack research across different plant species considering genotypes or plants with contrasting Cd tolerance. Increasing the pool will afford a look at species-specific and shared patterns of stress responses. Such a wider comparison may give indications for more focused breeding approaches and strategies and identify key regulatory pathways operating across plant species.
5. Overall, the integration of plant physiology, nutrient accumulation, and molecular responses has provided a panoramic view—from cellular level to phenotype—of how plants respond to Cd stress. With such insight, future studies will appropriately follow through on the translation gap from basic findings to field-level applications that will potentially lead to the development of crops with improved Cd tolerance, as well as to other environmental stressors.
6. The identification of specific phosphorylation sites in stress-related proteins opened new perspectives on plant defense mechanisms and furthered the impulse on research that should concentrate on the identification of more phosphorylation sites and their functional significance in stress adaptation as a means of further exploiting these mechanisms to enhance Cd tolerance.
7. Identifying potential solutions to Cd stress through integrated multi-omics involving, for instance, phosphoproteomics, transcriptomics, metabolomics, along with other omic approaches, will provide more functional details on how plants cope with Cd toxicity; all these data will be useful in breeding programs oriented to obtain tolerant plants to Cd contamination conditions.
8. Field-level considerations, phosphoproteomic comparisons involving both short- and long-term Cd exposure settings, and interactions among the various types of stresses represent targeted approaches. It must be underlined that, in the field, stresses due to Cd are not usually incurred alone but, rather, together with other abiotic and biotic stresses, including drought and pathogen attacks. Future studies will be required to understand how co-occurring stresses interact with Cd responses at the phosphoproteomic level. A proper understanding of such complex interactions among multiple stresses will help in fine-tuning strategies for Cd mitigation, covering both phytoremediation and environmental safety purposes. Among challenges to date is our ability to translate fundamental knowledge on phosphoproteomic response to Cd stress into practical field performance. What will be needed is to strike a balance between understanding phosphoproteomics and creating plant genotypes that are pleasing to agronomists. Further confirmation through field trials will be necessary to ascertain whether this indeed translates the findings in the laboratory into improved performance of crops in the field. Full implementation of phosphoproteomic findings will necessitate a serious commitment to closing the gap between laboratory and field applications. This will involve the validation of phosphoproteomic insights under varied environmental conditions, which would ensure that stress responses identified under the complex realities of agricultural systems are effective. Indeed, this will be an important way to develop robust crops with the capability to withstand Cd stress in different environments. By addressing these directions, future research would provide further insight into Cd stress responses, integrate omics with physiological understanding, and offer practical applications to enhance crop resilience in Cd-contaminated environments.
9. Different phosphoproteomic approaches and strategies have been used in the studies on the response of plants to Cd stress. We utilized a liquid chromatography–tandem mass spectrometry (LC–MS/MS)-based high-throughput label-free quantitative approach to identify, quantify, and compare phosphopeptides between Cd-tolerant and sensitive tomato genotypes [11]. Labeling with tandem mass tags (TMT), enrichment through high-pH reverse-phase high-performance liquid chromatography (HPLC), and analysis by LC-MS/MS were employed for seashore paspalum plants under Cd treatment [26]. Labeling with TMT, IMAC (immobilized metal affinity chromatography), fractionation using HPLC, and analysis by LC-MS/MS have been employed for poplar plants treated with Cd and N [27,28]. Fang et al. [25] used 2D gel electrophoresis followed by Matrix-Assisted Laser Desorption/Ionization Time-of-Flight/Time-of-Flight Tandem Mass Spectrometry (MALDI-TOF-TOF MS/MS) for analyzing differential accumulation of phosphorylated proteins in rice lines with contrasting Cd tolerance. Zhong et al. [24] adopted an iTRAQ (isobaric tag for relative and absolute quantitation)-based quantitative phosphoproteomics approach for the investigation of rice seedling responses under Cd exposure. Further improvement of phosphoproteomic technologies in the future would go towards enhancing the sensitivity to detect low abundance phosphopeptides, improving the enrichment methods of phosphopeptides, and/or developing bioinformatics tools for analyses and interpretation of the complex phosphoproteomic data. Future improvements will allow for an in-depth understanding of the role of protein phosphorylation in plant Cd stress responses and related biological processes. In addition, some potential approaches could be incorporated into such omics perspectives, such as Nuclear Magnetic Resonance (NMR) spectroscopy techniques, which can contribute to different stages of protein analysis, including characterizing the structure, dynamics, and interactions of proteins [43].
10. Our research group is currently working on a grafting technique—a practice with numerous basic plant physiology and real field applications, as performed and reviewed by our research group [1,2,44,45,46]—by combining phosphoproteomic analysis with physiological approaches. We expect that the grafted plants will provide new insights regarding differential Cd tolerance, probably by enhancing root-to-shoot communication and stress signaling. Preliminary results are encouraging, and we hope to present the results about phosphoproteome changes in grafted plants in forthcoming communications.

## Figures and Tables

**Table 1:** Experimental conditions along with exemplary findings in phosphoproteomic studies on cadmium exposure in plants.

Plant Species	Cd Treatment Used for Phosphoproteomic Analyses	Growth Medium	Duration of Cd Treatment	Plant Age	Plant Organs Used for Phosphoproteomic Analyses	Examplary Findings in Investigation	Reference
*Solanum lycopersicum*	35 µM CdCl_2_	Hydroponic solution	4 days	20-day-old seedlings	Leaves	Cd tolerance-related proteins (e.g., ABC transporters, HSPs), auxin signaling changes	[11]
*Oryza sativa*	10 µM and 100 µM (CdCl_2_.2.5H_2_O)	Hydroponic solution (Hoagland’s solution)	12 days	Seedlings after first leaf fully expanded	Shoots	Differential regulation of proteins related to signaling, stress tolerance, oxidative stress tolerance, and transcription factors	[24]
*Oryza sativa*	0.1 mmol/L CdCl_2_	Hydroponic solution	7 days	Third-leaf stage	Leaves	Differential accumulation of proteins related to carbon metabolism, signal transduction, and gene regulation	[25]
*Paspalum vaginatum*	500 µM CdCl_2_	1/2 Hoagland’s nutrient solution	72 h	30-day-old plants	Roots	Increased phosphorylation of PvFBA1, upregulated activity of pentose phosphate pathway (PPP) pathway, enhanced soluble sugar content	[26]
*Populus yunnanensis*	40 mg Cd/kg soil	Soil in pots	60 days	1-year-old seedlings	Leaves	Increased sucrose and soluble sugar; reduced ROS and membrane damage	[27]
*Populus yunnanensis*	1.22 g CdCl_2_.2.5H_2_O (40 mg Cd^2+^. Kg^−1^)—added to each pot every 5 days	Soil in pots	60 days	20 cm height (with 10–15 leaves)	Leaves	Increased Cd uptake and translocation; enhanced GSH and PCs levels; reduced H_2_O_2_ and MDA	[28]

## Data Availability

No new data were created or analyzed in this study. Data sharing is not applicable to this article.
